# Evidence for the Important Role of Oxidative Stress in the Pathogenesis of Acne

**DOI:** 10.31661/gmj.v0i0.1291

**Published:** 2019-04-10

**Authors:** Sina Kardeh, Seyed Arman Moein, Mohammad Reza Namazi, Bahareh Kardeh

**Affiliations:** ^1^Student Research Committee, Shiraz University of Medical Sciences, Shiraz, Iran; ^2^Cellular and Molecular Medicine Student Research Group, Medical School, Shiraz University of Medical Sciences, Shiraz, Iran; ^3^Molecular Dermatology Research Center, Shiraz University of Medical Sciences, Shiraz, Iran

**Keywords:** Acne Vulgaris, Oxidative Stress, Reactive Oxygen Species, mTOR, PPAR, Inflammation

## Abstract

Acne vulgaris is a common inflammatory skin disorder which is recognizable by dermatological lesions and scars. In addition to some pathogenetic factors such as hyperkeratinization, upregulated sebum secretion, and immunoinflammatory reactions, recent studies have also connected oxidative stress to the pathogenesis of acne vulgaris. In this article, we will briefly review clinical studies that interrogated alterations in oxidative stress biomarkers by a systematic search conducted in PubMed, Web of Science, and Scopus using "acne", "oxidative stress", and "reactive oxygen species" keywords. Overall, studies have shown that oxidative biomarkers (e.g. lipid peroxidation final products) are higher in acne vulgaris lesions. A significant positive correlation has also been noted between acne severity and oxidative biomarkers. In contrast, diminished levels of antioxidant enzymes (e.g. superoxide dismutase and catalase) have been observed in acne. We propose four probable mechanisms for the role of reactive oxygen species (ROS) in acne pathogenesis. We believe that ROS can contribute significantly to the acne vulgaris pathobiology via toll-like receptor (TLR), peroxisome proliferator-activated receptor (PPAR), mTOR pathway, and innate immune system, resulting in inflammation by alterations in the generation of several proinflammatory cytokines including IL-1, IL-8, and TNF-α.

## Introduction

Acne vulgaris is a self-limiting, chronic, inflammatory skin disorder [1-3]. It is believed that almost everyone experiences different stages of acne vulgaris skin manifestations at the onset of puberty and adolescence. In most cases, the severity of skin symptoms appears to decrease with increasing age [4]. In terms of etiology, four crucial factors are suggested to participate in acne pathology [5, 6]:

*1.Propionibacterium acnes* which is responsible for the production of proinflammatory mediators by the immune system

2.sebaceous gland hypersecretion of sebum

3.hyperkeratosis followed by obstruction of the follicle

4.inflammatory factors produced by the skin and immune system

Previously, it has been advocated that oxidative stress might be engaged in skin disorders such as chronic ulcers [7], allergic reactions [8], and vitiligo [9]. Recent studies have focused on the essential relationship between oxidative stress and the pathology of acne vulgaris [10-13]. It is believed that an imbalance in the production of oxygen-derived pro-oxidants, also known as reactive oxygen species (ROS), and cellular capacity of antioxidant defense, probably leads to oxidative stress phenomenon and augmentation of effects of ROS. The ROS family comprises free radicals such as nitric oxide radical (NO), superoxide ion radical (O2-), hydroxyl radical (OH) and non-radicals such as hydrogen peroxide (H2O2) and ozone (O3) which have been implicated in mutation, carcinogenesis, inflammation and aging [14-16]. Moreover, interactions between ROS and lipids result in polyunsaturated fatty acid peroxidation and production of toxic aldehydes such as malondialdehyde (MDA), which can be used as biomarkers to evaluate lipid peroxidation in cells [17]. The detection and quantification of MDA are pivotally performed by assay of thiobarbituric acid reactive substances (TBARS) since interactions of thiobarbituric acid with MDA creates an easily detectable pink color which can be measured by spectrophotometry [18]. In addition to the toxic features of ROS, it is believed that accumulation of ROS such as the production of H2O2 from the neutrophils has other unfavorable effects including inflammation and tissue damage [19].

P. acnes produces a variety of chemical factors that induce neutrophil chemotaxis while neutrophils attempt to attack P. acnes by ROS secretion, which can initiate inflammation in normal tissues leading to acne skin symptoms [20, 19]. Many enzymes and compounds such as catalase (CAT), superoxide dismutase (SOD), myeloperoxidase (MPO), glutathione (GSH), and xanthine oxidase (XO) are involved in the antioxidant protection system and remove detrimental ROS compounds by converting them into non-toxic molecules like H2O or by processing the ROS molecules into substrates of the other antioxidant enzymes. In this article, we review the current literature that corroborates the involvement of oxidative stress in the pathophysiology of acne vulgaris and discuss the potential mechanisms that might ultimately link ROS overproduction to the clinical manifestations of this disease.

### Search Strategies

**Figure 1:**
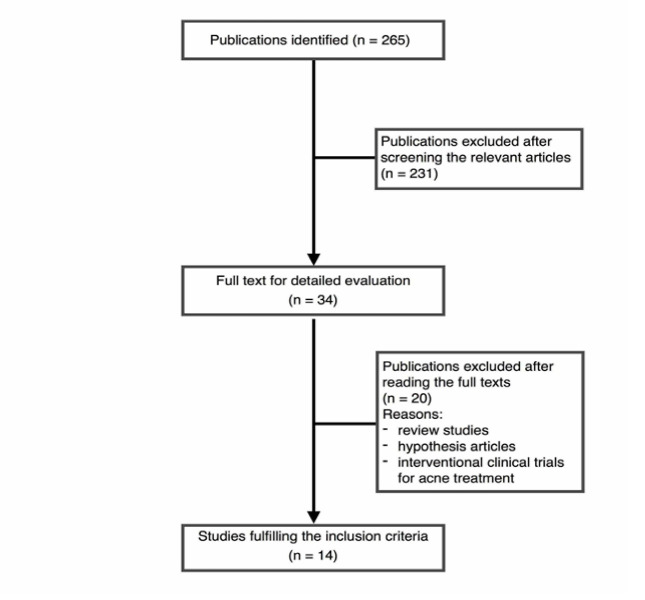
The process of including and excluding studies

This systematic search was conducted in English literature to investigate evidence measuring oxidative stress in acne vulgaris patients. Databases including PubMed, Web of Science and Scopus were searched within a time limitation from 1980 up to July 2018. The search terms in the title and abstract were “oxidative,” “oxidant,” “antioxidant,” “antioxidative,” “redox,” “reactive oxygen species,” “ROS,” and “free radicals,” while “acne” was searched in the whole text. Keywords were selected based on MeSH (Medical Subject Headings), which is a controlled vocabulary thesaurus used for indexing articles published in PubMed. We included any clinical observational study that examined skin or serum levels of oxidant or antioxidant biomarkers in acne patients regardless of the severity of acne and presences of a control group. A modified version of the Grading of Recommendations Assessment, Development and Evaluation Working Group (GRADE) approach was used for quality assessment [21]. There were no restrictions on study design and the method of assessing oxidative biomarkers. Exclusion criteria were case reports, older studies (before 1980) and publications without available full texts. In the first step, a single reviewer screened 265 articles retrieved from the searches based on article title and abstract. This process led to the exclusion of 231 irrelevant articles. Of the 34 remaining articles, 14 met our inclusion criteria and were selected for data collection (Figure-1). Furthermore, to ensure that no other potentially relevant articles were missed, references of all included studies were checked after retrieving their full texts. The included clinical articles were reviewed to extract the type of sampling, study population and grouping, evaluated biomarkers and their level (Table-1).

## Results

A summary of study designs and key findings of articles are presented in Table-1. The investigations differ in methodology regarding sampling, grouping, and selection of biomarkers of oxidative stress.

## Discussion

This review was performed to shed more light on the role of ROS in acne pathogenesis. Acne vulgaris is traditionally believed to result from four crucial factors including hyperkeratinization, upregulated sebum secretion, immune and inflammatory reactions, and follicle colonization by P. acnes [6]. A considerable number of acne vulgaris therapeutic agents are designed for management of these four classical factors. Our review shows the role of ROS in the pathogenesis of this disorder. Recent studies suggest that various mechanisms and molecular pathways link oxidative stress to the pathogenesis of acne vulgaris:

### Toll-Like Receptors

Toll-like receptors (TLRs) are a group of transmembrane glycoproteins consisting of extra- and intracellular pattern recognition receptors that are well known for their defensive responsibilities in diverse cell types such as keratinocytes and Langerhans cells in the skin and monocytes, macrophages, dendritic cells, mast cells, and lymphocytes among the immune system cells [22]. TLRs have been suggested to induce production of pro-inflammatory factors in the human body [23]. Considering the significant roles of pro-inflammatory factors in acne vulgaris inflammation, in 2002, Kim *et al* . revealed that P. acnes Lipopolysaccharides (LPS) potently provoke TLR2 in monocytes which is followed by TNF-α, IL-1β, and IL-8 secretion that act as chemoattractant for other immune cells [24]. In 2012, Dispenza *et al* . reported a marked increase in TLR2 production via P. acnes LPS in acne patients’ monocytes comparing to the healthy control group’s monocytes [25]. Moreover, in 2013, Selway *et al* . observed a significant TLR2-activated production of IL-1α from the primary human keratinocytes explaining the reported high level of IL-1α in acne sites [26, 27]. Surprisingly, TLR has been implicated as a ROS production inducer in various cells such as dendritic cells [28], PBMCs [29], and macrophages [30]. Furthermore, it is believed that LPS-activated TLR regulated ROS generation is conducted via activation of NADPH oxidase [31, 32]. It is noteworthy to mention that oxidative stress and subsequent inflammation observed in acne vulgaris might result from activation of TLR receptors in various cells in the skin by P. acnes LPS.

### PPAR

Peroxisome proliferator-activated receptors (PPARs) belong to a family of ligand-activated nuclear transcription factors highly expressed in fatty acid catabolic organs. PPARβ/δ, which is assumed to be the crucial regulator for important functions such as cell proliferation and differentiation, is markedly available in the skin among the various isotypes [33]. However, in sebocytes, it is believed that PPARα and PPARγ are the key mediators of lipid metabolism [34]. Moreover, PPARα and γ appeared to be triggered by products of 5-lipoxygenase (5- LOX) which is also believed to be upregulated in acne vulgaris [35], and some studies have shown that 5-lipoxygenase inhibitors such as zileuton potently diminish acne vulgaris clinical manifestations [36]. It seems that 5-LOX activation is followed by production of inflammatory factors such as IL-6 and IL-8 in acne patients [37]. Studies have suggested that PPAR can be potently triggered by lipid peroxidation, as a major consequence of molecular damage induced by oxidative stress in acne vulgaris pathology [38]. Oxidative stress mainly causes the production of lipids from the sebocytes via PPARγ [35]. Considering the significant roles of PPARs in the induction of lipid production and inflammation in sebocytes, the involvement of PPARs in acne disorder seems undeniable.

### Innate Immune System

P. acnes has the potential to onset a set of reactions in the skin which results in the production of IL-1α, IL-8, and TNF-α from skin cells such as sebocytes and keratinocytes, in response to its microbial compounds such as LPS. Besides the general inflammatory effects of these molecules, IL-8 can profoundly trigger neutrophil chemotaxis which is followed by secretion of a marked amount of ROS by neutrophils in order to destroy P. acnes [39]. Moreover, neutrophils of acne patients appear to secrete more hydrogen peroxide compared to the normal neutrophils [19]. As an off-target of the neutrophil-derived ROS attack and the following lipid peroxidation, the follicular wall is demolished, triggering the expression and secretion of more pro-inflammatory factors such as IL-1α [40, 41]. Furthermore, IL-1 can induce endothelial [42] and polymorphonuclear leukocytes [43] to secrete ROS (predominantly O2-) causing further changes and damages in the structure of the surrounding tissues and leading to inflammation. Interestingly, these findings are not significant only theoretically, since the anti-acne effect of cyclines such as clindamycin and tetracycline is believed to be achieved mainly through diminishing neutrophil-derived ROS [44, 45].

### mTOR

As a member of phosphatidyl 3-kinase (PI3K)-related kinase protein family, the mechanistic target of rapamycin (mTOR) is an evolutionarily-conserved serine/threonine kinase that in response to a variety of extracellular cues from nutritional status, growth factors, and stress signals acts as a central regulator of cell metabolism, growth, proliferation, homeostasis and survival [46]. Since this pathway regulates many major cellular processes and mTOR overactivation has been implicated in various health disorders including aging, obesity, type 2 diabetes, and also acne, recently several studies have been dedicated to developing drugs that can target this enzyme [47-49]. Previously, clinical investigations have linked low insulin sensitivity and high glycemic load diet, which can augment insulin/insulin-like growth factor-1 (IGF-1) signaling, with the pathogenesis of acne [50, 51]. The forkhead box transcription factor O1 (FoxO1) and the mTOR complex 1 (mTORC1) are the predominant mediators in processing cellular nutritional status [52].

Further, FoxO1 and mTORC1 interact with various crucial pathways of sebaceous gland homeostasis including sebaceous gland hyperplasia and lipogenesis, androgen signaling, the activity of innate and adaptive immunity and also inflammatory responses such as TNF-α, which all converge in acne pathophysiology [53]. As mTOR is a key player in cell metabolism and energy balance, its effects on ROS level are of significant importance. So far, various studies have evaluated the synergistic expression of mTOR and ROS escalation in vitro and in vivo. Although there is no doubt that different concentrations of ROS can regulate mTORC1 activity, the relationship between oxidative stress and mTOR seems to be highly dependent on microenvironmental context and ROS dosage. Recent studies demonstrated high levels of mTOR and FoxO1 expression in skin biopsies taken from acne patients concomitant with increased IGF-1 serum level [54, 55].Also, there is direct evidence for deviated FoxO1/mTORC1 signaling in the induction of acne vulagarissebofollicularinflammasomopathy [56], which is a key player in the initiation of oxidative mediated pathways. Hence, agents that can target mTOR are potent anti-acne therapeutic options that act via multiple pathways of acne pathomechanism.

## Conclusion

This study reviews the evidence for the pivotal role of oxidative stress in the pathogenesis of acne vulgaris and suggests that oxidative stress can considerably contribute to the pathobiology of acne vulgaris via various pathways including PPARs, TLRs, mTOR and innate immune system. This review can stimulate further research on the therapeutic effect of anti-oxidants against this very common disorder.

## Conflicts of Interest

There are no conflicts of interest.

**Table 1:** Clinical studies on the activity of oxidant or antioxidant biomarkers in acne patients

**Year/** **Authors**	**Sampling/ study population**	**Grouping**	**Evaluated biomarkers**	**Main Findings**
2018, Awad *et al* (21)	60 patients, 40 control, Blood sampling	Mild (9), moderate (33), server (18), control (40)	MDA, TAC	Acne patients showed higher serum MDA and lower TAC compared with control subjects No significant difference was detected in MDA, and TAC level with respect to acne severity
2014, Al-Shobaili (10)	156 patients, 47 control, Blood sampling	Mild (46), moderate (83), severe (27), control (46)	CAT, SOD, TAC, MDA.	CAT, SOD, and TAC were significantly higher in the control group, MDA was significantly higher in the patients, No significant difference was reported in CAT activity between the mild, moderate, and severe acne patients, SOD was significantly lower and TAC and MDA were significantly higher in severe acne compared to mild and moderate groups.
2014, Yang *et al* (13)	50 patients, Skin biopsies from chin, cheek, and forehead	Nonsmoker (22), former smoker (7), Current smoker (21) (smokers n=28)	LPO	LPO level was significantly higher in the smokers, No significant difference was observed between the current and former smokers, No significant difference was observed in the LPO content of different facial area comedones and lesions in the acne patients.
2013, Al-Shobaili *et al* (11)	50 patients, 40 control, blood sampling	Mild (20), moderate (23), severe (7), control (40)	GSH, NO, SOD, MDA, Protein oxidation	NO, MDA, and protein oxidation were significantly elevated in patient group, GSH and SOD were markedly diminished in the patient group, Overall, SOD and GSH negatively, and MDA, NO and protein oxidation positively correlate with severity of acne vulgaris.
2012, Perihan *et al* (12)	50 patients, 40 control, Skin scraping from face	Mild (16), Moderate (18), Severe (16), control (40)	GSH, MDA, SOD, CAT	SOD, CAT, and GSH negatively, and MDA positively correlate with the severity of acne vulgaris.
2011, Ikeno *et al*(22)	40 patients, 19 control, biopsy from involved and uninvolved face and upper arm, female study population	Patient (40), Control (19)	GSH	GSH level was significantly lower in patients, GSH level of upper arm skin was significantly higher than face skin, No significant difference was observed between the diseased and healthy skin of the patients.
2010, Sarici *et al* (1)	32 patients, 34 control, blood sampling	Patient (32), Control (34)	MDA, SOD, CAT, NO, XO	MDA serum level and XO activity were significantly increased in the patients, SOD and CAT activity were significantly reduced in the patients, The serum level of NO was not markedly different between the control and patient groups.
2009, Abulnaja (23)	60 patients, blood sampling, female study population	Obese with acne (15), Obese without acne (15), Non-obese with acne (15), Non-obese without acne (15)	Vitamin A, Vitamin E, Vitamin C, MDA, β-carotene, MAO,	Vitamin A, E, C content was diminished in the acne patients, vitamin C was markedly decreased in obese patients compared with non-obese patients, MDA positively whereas MAO and β-carotene negatively correlate with obesity and acne severity.
2008, Fattah *et al* (24)	23 patients, 23 control, Blood sampling, skin biopsy	Mild (6), Moderate (10), Severe (7), Control (23)	SOD, MDA	SOD activity and MDA content were upregulated in the patients, SOD activity negatively and MDA content positively correlated with acne severity, SOD activity and MDA content of the skin biopsies were drastically higher compared to the blood sample.
2005, Kurutas *et al* (25)	43 patients, 24 control, Blood sampling,	Mild (7), Moderate (31), Severe (5), Control (24)	MPO, SOD	SOD activity was significantly lower in polymorphonuclear leukocytes of the patient group, no marked difference was reported between MPO activities of the patient and control group.
2005, Arican *et al* (26)	43 patients, 46 control, Blood sampling,	Patient (43), Control (46)	MDA, SOD, G6PD, CAT	MDA and SOD levels were significantly escalated in acne patients, G6PD and CAT levels were significantly lower in acne patients.
2005, Aybey *et al* (27)	79 patients, 17 control, Blood sampling,	Smokers (11), Nonsmokers (68), Control (17)	GSH-Px	GSH-Px level was significantly lower in the patients, no marked difference was observed in the GSH-Px level among smoker and non-smokers, No correlation was reported between the acne severity and GSH-Px level.
2001, Basak *et al* (28)	52 patients, 36 control, Blood sampling,	Mild (23%), Moderate (75%), Severe (2%), Control (n=36)	GSH-Px, SOD, CAT,	SOD and GSH-Px activity were significantly reduced and CAT activity and TBARS level were increased in acne patients,
1984, Michaelsson and edqvist (29)	89 patients,	severe male patients (n=47), moderate female patients (n=26), severe female patients (n=21).	GSH-Px	GSH-Px level was reported to be significantly diminished in the male patients comparing to the control, No significant difference was observed in GSH-Px level of female subjects comparing to the control group,
